# Mini-Basketball for Preschool and School-Aged Children with Autism Spectrum Disorder: A Systematic Review of Randomized Controlled Trials

**DOI:** 10.3390/healthcare13222861

**Published:** 2025-11-11

**Authors:** Daniel González-Devesa, Rui Zhou, Markel Rico-González, Carlos D. Gómez-Carmona

**Affiliations:** 1 Research Group on Physical Activity, Education, and Health (GIAFES), Catholic University of Ávila, 05005 Ávila, Spain; 2 School of Sports Management and Communication, Capital University of Physical Education and Sports, Beijing 100191, China; 3 Department of Didactics of Music, Plastic and Body Expression, University of the Basque Country, UPV-EHU, 48940 Leioa, Spain; 4 Research Group in Training, Physical Activity and Sports Performance (ENFYRED), Department of Music, Plastic and Body Expression, University of Zaragoza, 44003 Teruel, Spain; carlosdavid.gomez@unizar.es; 5 Research Group in Training Optimization and Sports Performance (GOERD), University of Extremadura, 10005 Caceres, Spain; 6 BioVetMed & SportSci Research Group, University of Murcia, 30001 Murcia, Spain

**Keywords:** executive function, exercise therapy, psychomotor performance, team sports, youth sports

## Abstract

Background: Although the participation of children with autism spectrum disorder (ASD) in team sports presents challenges, group-based physical activities could offer specific benefits for their core symptoms. Therefore, the aim of this systematic review was to analyze the benefits of mini-basketball for children with ASD. Methods: A systematic review was conducted following PRISMA guidelines and was registered in PROSPERO (CRD420251144800). Four databases (Web of Science, SPORTDiscus, PubMed, and Scopus) were searched to select randomized controlled trials reporting the effects of mini-basketball interventions on children with ASD from their inception to August 2025. Results: Eight randomized controlled trials involving 436 participants (aged 3–12 years, 87.3% male) met the inclusion criteria. All studies were conducted in China using 12-week interventions (40–45 min, 2–5 days/week at moderate intensity). The quality was rated as good in two studies and fair in six. Five studies assessed social responsiveness, with four showing significant pre–post reductions in the experimental groups and all demonstrating superior outcomes versus those of the controls. One study reported significant reductions in repetitive behaviors, self-injurious behaviors, and restricted behaviors compared to that of the controls. Joint attention improvements were observed through eye-tracking measures, with increased fixation counts, shorter time to first fixation, and more accurate gaze shifts. Physical fitness benefits included improved shuttle run times and standing long jump performance. Finally, one study demonstrated better inhibition control and improvements in sleep quality, including increased sleep duration and efficiency. Conclusions: Mini-basketball interventions can improve social responsiveness and related outcomes in children with ASD. These findings support mini-basketball as a feasible, safe, and effective intervention that could be integrated with existing therapeutic approaches.

## 1. Introduction

Autism spectrum disorder (ASD) is a neurodevelopmental condition diagnosed based on behavioral characteristics observed during childhood [1]. Although the term “spectrum” is used because it affects each person differently, the behavioral characteristics associated with this diagnosis include difficulties with social interaction and communication, as well as restricted and repetitive behaviors and interests [2]. These characteristics derive from various neuroanatomical alterations, such as modified connectivity patterns, region-specific volumetric changes, variations in gray or white matter, and fine microstructural or cellular modifications [3,4]. In order to mitigate its consequences, different pharmacological treatments have been evaluated to address comorbidities, particularly concurrent mental health symptoms (e.g., irritability, anxiety, and inattention/hyperactivity, among others) [5,6,7]. However, it remains essential to explore non-pharmacological interventions that directly address the core symptoms (social communication and restricted/repetitive behaviors) [8].

Among other non-pharmacological treatments, physical activity (PA) has been highlighted as one interesting way to treat mental issues, including ASD [9,10]. Following different meta-analytical comparisons, it can be found that PA has reported positive effects on motor skills [11], sleep-related issues [12,13], and/or cognitive functions (i.e., memory, attention, executive functions) [14,15]. However, there is a significant body of literature reporting the positive effects of PA on core symptoms (i.e., social disorder and repeated rigid behavior) of children with ASD diagnosis [16]. Specifically, there are meta-analyses reporting that physical exercise as an effective tool for reducing the number of episodes of stereotypical behaviors [17] and for enhancing communication and social interaction in children with ASD diagnosis [18,19]. Interestingly, there are also different meta-analytical comparisons that, after clustering the scientific research focusing on improving ASD core symptoms, have highlighted that group-based activities are of particular interest [20,21].

Considering this idea, the concept of involving children with ASD diagnoses in team sport-based activities is of interest. However, when a group-based multiple trajectory modeling approach has been used to analyze trajectories of participation in team and individual sports, outcomes have reported that involving children in team sports is challenging [22]. Specifically, the results of this study highlight that children with ASD diagnoses used to belong to the “sports avoider” group, and they are particularly likely to experience exclusion from team sport environments, and this exclusion persists over time [22]. However, although for people with an ASD diagnosis, playing team sports means dealing with difficulties regarding social interactions and limitations in motor functions, some researchers have decided take advantage of the opportunity to improve autism core symptoms [23] by supporting the use of these sports when working with young people with ASD diagnoses. In fact, although a large body of literature is lacking, different articles have reported the results. For example, participation in a community organized football program can improve social interactions, behavioral functioning, and communication skills in children up to the age of 12 years old [24,25]. However, since it appears that there are more intervention programs based on basketball, it seems that this team sport is of particular interest to research teams [26].

Compared to typically developing children, children with ASD exhibit significant inefficiencies in regards to motor tasks such as hand–eye coordination, throwing, and catching, suggesting difficulties in motor control and perceptual–motor integration [27]. At the same time, organized physical activity (OPA) in group settings has been shown to effectively promote social interaction and communication skills in children with ASD [21].

Against this backdrop, basketball—a team sport that requires active upper-limb involvement, relies on fine hand–eye coordination, and demands continuous visual information processing—holds unique potential. Mini-basketball, in particular, is an adapted form of the sport designed for children, using lighter and smaller equipment, along with developmentally appropriate modifications of rules and court size [28]. This structured, low-intensity, and highly interactive format preserves the cooperative and competitive nature of traditional basketball while enhancing playfulness and accessibility. It allows children with ASD to participate in training safely and consistently at a suitable pace. Existing studies have demonstrated that mini-basketball significantly improves fundamental motor skills, physical fitness, and social communication abilities in children with ASD [29].

For all these reasons, looking for the effects of team sports, in general, and basketball, in particular, on children with an ASD diagnosis could be of interest. To the best of the authors’ knowledge, there is no systematic review attempting to analyze all the effects of a certain team sport on children with an ASD diagnosis. The most relevant articles include the following: first, a systematic review and meta-analysis that analyzed the effects of basketball on executive function in children with an ASD diagnosis, reporting that mini-basketball is outstanding for improving inhibitory control and cognitive flexibility [26], and second, a narrative review aimed at reporting the effects, improvements, and difficulties experienced by people with autism when playing soccer [23]. For this reason, this systematic review aims to analyze the benefits of basketball for children diagnosed with ASD, both in relation to core symptoms and other associated outcomes, based on randomized controlled trials (RCTs). This review may be of interest to professionals working with children with an ASD diagnosis, either in school settings or in extracurricular activities, providing a possible way to help mitigate symptoms and enhance quality of life.

## 2. Materials and Methods

The present systematic review was conducted in accordance with the Preferred Reporting Items for Systematic Reviews and Meta-Analyses (PRISMA) guidelines (Supplementary Material File S1) [30] and guidelines for performing systematic reviews in sport sciences [31]. The review protocol was registered in PROSPERO (CRD420251144800).

### 2.1. Search Strategy

Systematic searches were conducted in four electronic databases—Web of Science, SPORTDiscus, PubMed, and Scopus—from their inception to 29 August 2025. An initial exploratory search was carried out to identify the Boolean search string most appropriate for this review. Table 1 presents the search strategy used in each database. Moreover, the reference lists of the included studies, as well as citations identified via Google Scholar, were examined to identify additional studies for potential inclusion in the present review.

### 2.2. Eligibility Criteria

Studies were only eligible for inclusion if they conducted RCTs that reported the effects of mini-basketball interventions in children with ASD. Studies were excluded if: (a) mini-basketball was combined with other exercises or therapies; (b) they included both children and adolescents and did not report results for children separately; (c) the intervention consisted of a single session; or (d) the full text was unavailable. Only studies published in English, Spanish, or Portuguese were included.

### 2.3. Study Selection and Data Extraction

Duplicate references were removed manually using Rayyan (version 1.5.0; QCRI, Doha, Qatar) [32] prior to screening. The titles and abstracts of all identified studies were independently evaluated by both authors (D.G.-D. and C.D.G.-C.) to determine eligibility. After this initial screening, the selected studies were reviewed in full by both researchers (D.G.-D. and C.D.G.-C.) to confirm inclusion. Any disagreements were resolved through discussion and consensus. Full-text articles deemed potentially relevant were retrieved for further assessment.

From each included article, data were extracted on the title, authors, year of publication, country, participant characteristics, intervention characteristics, outcomes assessed, main results, dropouts, and adverse events. One researcher performed the data extraction, and a second researcher verified its accuracy.

### 2.4. Quality Assessment

Methodological quality for each RCT was obtained from the Physiotherapy Evidence Database (PEDro), when available. If a trial was not listed in PEDro, two authors (D.G.-D. and C.D.G.-C.) independently assessed its quality and resolved discrepancies through consensus. Quality was classified using the following cutoffs: excellent (9–10), good (6–8), fair (4–5), and poor (<3) [33].

## 3. Results

### 3.1. Study Selection and Screening Results

**Figure 1:**
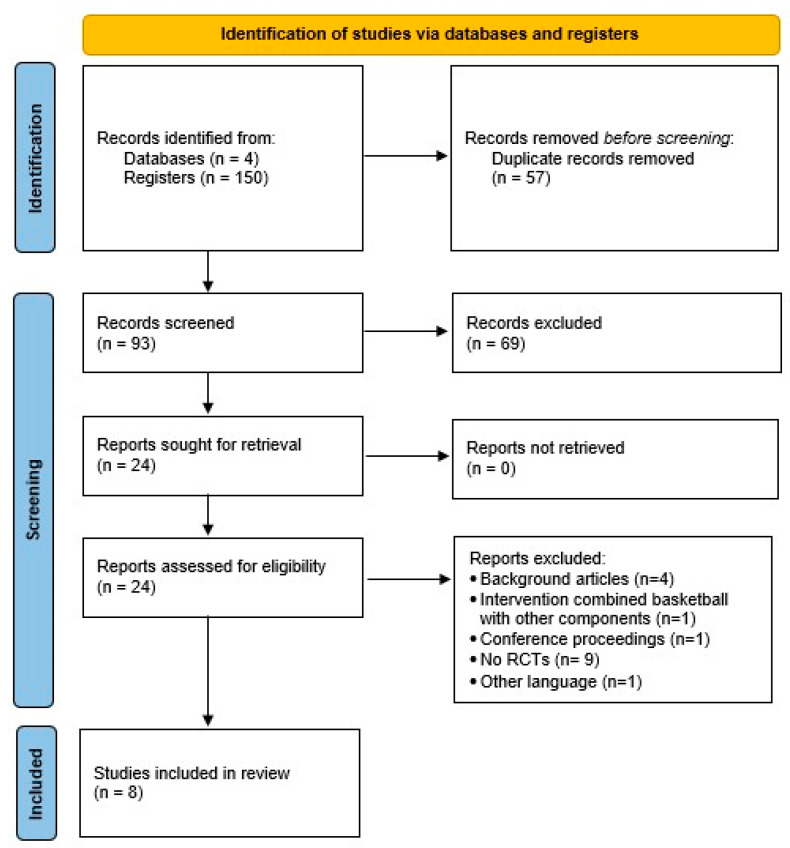
PRISMA (Preferred Reporting Items for Systematic Reviews and Meta-Analyses) study flow diagram.

Through the database search, a total of 150 records were identified. After removing duplicates, 93 records remained for title and abstract screening. Following this process, 24 articles were selected for full-text review. Finally, eight RCTs [34,35,36,37,38,39,40,41] met the inclusion criteria and were included in the present systematic review (Figure 1).

### 3.2. Quality Assessment Results

The methodological quality of the reviewed RCTs was rated as “good” in two studies [40,41] and “fair” in the rest of the selected studies (Table 2). The blinding of participants and therapists was the most relevant methodological issue not adequately addressed in the studies, which is common in this type of intervention. Other frequent shortcomings included the lack of key outcomes measured in more than 85% of participants, the absence of intention-to-treat analysis, and the lack of concealed allocation.

### 3.3. Study Design and Samples

The combined sample size across the reviewed studies was 436 participants (Table 3). The study with the smallest sample included 40 participants [36], while the largest comprised 63 participants [35]. Demographic characteristics varied, with ages ranging from 3 to 12 years old, situating the sample within a child population (from preschool to preadolescence). Among studies that reported sex in the final samples (*n* = 260), 227 participants were male (87.3%). Overall, the studies were published between 2019 and 2025, and all were conducted in China.

### 3.4. Characteristics of Interventions

All interventions lasted 12 weeks across the included studies. Session duration consistently ranged from 40 to 45 min. A five-days-per-week schedule was used in seven of the eight trials. Only one study followed a different weekly frequency, employing two sessions per week [40]. Exercise intensity, reported in six studies, was typically described as moderate (most often operationalized at 128–148 bpm), with one trial prescribing 60–69% of HR_peak_ [38]. Only two studies did not report intensity [37,40]. Control groups maintained their usual routines and did not receive any structured basketball training.

### 3.5. Main Results

#### 3.5.1. Social Responsiveness

Five studies assessed social responsiveness using the Social Responsiveness Scale, Second Edition (SRS-2). Four of the five trials reported significant pre–post reductions in SRS-2 total score for the basketball group [34,35,36,37], whereas all five trials observed increases in the control groups. After interventions, all trials reported lower SRS-2 total scores in the experimental group compared to controls, thereby supporting the superior efficacy of the basketball-based programs [34,35,36,37,38].

In the trial comparing mini-basketball with a ball-combination program (mini-basketball progressing to soccer), both intervention arms achieved pre–post reductions in the SRS-2 total score [35], and both active programs showed lower SRS-2 total scores than those of the controls.

**Table 3:** Descriptive characteristics of the included studies.

First Author (Year), Design	Sample	Intervention	Outcomes	Results	Dropouts and Adverse Events
Zhang et al. (2025) [39] Country: China	Participants (*n*): 57 (30EG; 27CON) Final sample (*n*): 28 (15EG; 13CON) Sex: M Age, *years* (mean; SD): EG: 4.90 ± 0.66 CON: 4.77 ± 0.70	Duration: 12 weeks Frequency: 5 days/week Volume: 40 min Intensity: Moderate EG: Mini-basketball program + ABA Activities: Typical exercise session had four sequential components, including introduction, warm-up activity, mini-basketball, and relaxation. CON: Maintained their regular routine + ABA.	Behavioral outcome measures: Repetitive behavior scale ■ Stereotyped behavior ■ Self-injurious behavior ■ Compulsive behavior ■ Ritualistic behavior ■ Sameness behavior ■ Restricted interests	Intra-group (*p* < 0.05): ↑ Repetitive behavior scale (total score) in *CON* ↑ Restricted behavior in *CON* Inter-group (*p* < 0.05): *EG* < Repetitive behavior scale (total score) than *CON* *EG* < Self-injurious behavior than *CON* *EG* < Restricted behavior than *CON*	Dropouts: 29 EG: 15 did not complete the experiment CON: 12 did not complete the experiment; 2 missing T1-MPRAGE data Adverse events: NR
Yang et al. (2024) [34] Country: China	Participants (*n*): 59 (30EG; 29CON) Final sample (*n*): 30 (15EG; 15CON) Sex: EG: 13M/2F CON: 13M/2F Age, *years* (mean; SD): EG: 5.17 ± 0.72 CON: 4.67 ± 0.70	Duration: 12 weeks Frequency: 5 days/week Volume: 40 min Intensity: Moderate; 128–148 bpm EG: Mini-basketball program + ABA Activities: Introduction, warm-up exercises, basketball intervention, and relaxation. CON: Maintained their regular routine + ABA.	Social Responsiveness: Social Responsiveness Scale, Second Edition ■ Social awareness ■ Social cognition ■ Social communication ■ Social motivation ■ Behavior pattern	Intra-group (*p* < 0.05): ↓ Social responsiveness (total score) in *EG* ↓ Social communication (score) in *EG* ↓ Behavior pattern (score) in *EG* ↑ Social responsiveness (total score) in *CON* ↑ Social communication (score) in *CON* ↑ Behavior pattern (score) in *CON* Inter-group (*p* < 0.05): *EG* < Social responsiveness (total score) than *CON* *EG* < Social cognition (score) than *CON* *EG* < Social communication (score) than *CON* *EG* < Behavior pattern (score) than *CON*	Dropouts: 29 EG: 14 did not complete behavioral assessment 1 Did not complete rs-fMRI scan CON: 13 did not complete behavioral assessment 1 Did not complete rs-fMRI scan Adverse events: NR
Qi et al. (2024) [35] Country: China	Participants (*n*): 63 (20EG1; 23EG2; 20CON) Final sample (*n*): 41 (13EG1; 14EG2; 14CON) Sex: EG1: 10M/3F EG1: 12M/2F CON: 13M/1F Age, *years* (mean; SD): 4.99 ± 0.76	Duration: 12 weeks Frequency: 5 days/week Volume: 40–45 min Intensity: Moderate; 128–148 bpm EG1: Ball combination training program + ABA Activities: Phases I–II: mini-basketball. Late Phase II: introduces soccer. Phase III: group games combining mini-basketball + soccer EG2: Mini-basketball program + ABA Activities: Exercise session had four sequential components, including introduction, warm-up, mini-basketball intervention, and cool-down. CON: Maintained their regular routine + ABA.	Social Responsiveness: Social Responsiveness Scale, Second Edition ■ Social awareness ■ Social cognition ■ Social communication ■ Social motivation ■ Behavior pattern	Intra-group (*p* < 0.05): ↓ Social responsiveness (total score) in *EG1* ↓ Social awareness (score) in *EG1* ↓ Behavior pattern (score) in *EG1* ↓ Social responsiveness (total score) in *EG2* ↓ Social communication (score) in *EG2* ↓ Social cognition (score) in *EG2* ↑ Social responsiveness (total score) in *CON* ↑ Behavior pattern (score) in *CON* Inter-group (*p* < 0.05): *EG1 and EG2* < Social responsiveness (total score) than *CON* *EG1* < Social awareness (score) than *CON* *EG1* < Behavior pattern (score) than *CON* *EG2* < Social communication (score) than *CON* *EG2* < Social cognition (score) than *CON*	Dropouts: 22 lost to follow-up EG1: 7 EG2: 9 CON: 6 Adverse events: NR
Ge et al. (2024) [41] Country: China	Participants (*n*): 49 (23EG; 26CON) Final sample (*n*): 30 (16EG; 14CON) Sex: EG: 15M/1F CON: 12M/2F Age, *years* (mean; SD): EG: 7.50 ± 2.19 CON: 6.64 ± 1.45	Duration: 12 weeks Frequency: 5 days/week Volume: 40 min Intensity: Moderate; 128–148 bpm EG: Mini-basketball program + ABA Activities: 10 min warm-up with short games, a 25 min combining mini-basketball skills and fitness, and a 5 min cool-down. CON: Maintained their regular routine + ABA.	Joint Attention: Joint attention task (Tobii Pro Fusion high performance portable eye tracker) ■ AOI Fixation duration ■ AOI Fixation count ■ AOI Visits count ■ Time to first fixation ■ Accurate gaze shifts count ■ Accurate-to-inaccurate gaze shift ratio	Intra-group (*p* < 0.05): ↑ Time to first fixation in *CON* ↓ Accurate gaze shifts count in *CON* Inter-group (*p* < 0.05): *EG* > AOI fixation count than *CON* *EG* < Time to first fixation than *CON* *EG* > Accurate gaze shifts count than *CON* *EG* > Accurate-to-inaccurate gaze shift ratio than *CON*	Dropouts: 19 EG: 1 did not complete post-test 3 missing MRI data 1 excessive head motion 2 missing behavioral data CON: 7 due to the impact of COVID-19 epidemic 4 missing MRI data 1 excessive head motion Adverse events: NR
Yu et al. (2021) [36] Country: China	Participants (*n*): 40 (20EG; 20CON) Final sample (*n*): 31 (17EG; 14CON) Sex: EG: 15M/2F CON: 13M/2F Age, *years* (mean; SD): EG: 4.89 ± 0.80 CON: 4.75 ± 0.62	Duration: 12 weeks Frequency: 5 days/week Volume: 40 min Intensity: 136.97 ± 7.45 bpm EG: Mini-basketball program Activities: Including a 2 min classroom routine preparation, then 8 min warm-up activities, followed by a 25 min mini-basketball training and a 5 min cool-down. CON: Maintained their regular routine.	Social Responsiveness: Social Responsiveness Scale, Second Edition ■ Social awareness ■ Social cognition ■ Social communication ■ Social motivation ■ Behavior pattern	Intra-group (*p* < 0.05): ↓ Social responsiveness (total score) in *EG* ↓ Social cognition (score) in *EG* ↓ Social communication (score) in *EG* ↑ Social responsiveness (total score) in *CON* ↑ Social communication (score) in *CON* ↑ Behavior pattern (score) in *CON* Inter-group (*p* < 0.05): *EG* < Social responsiveness (total score) than *CON* *EG* < Social communication (score) than *CON* *EG* < Social cognition (score) than *CON* *EG* < Behavior pattern (score) than *CON*	Dropouts: 9 6 missing MRI data 3 inferior images not suitable for analysis EG: 3 CON: 6 Adverse events: NR
Yang et al. (2021) [38] Country: China	Participants (*n*): 59 (30EG; 29CON) Final sample (*n*): 30 (15EG; 15CON) Sex: EG: 13M/2F CON: 12M/3F Age, *years* (mean; SD): EG: 4.67 ± 0.70 CON: 5.03 ± 0.55	Duration: 12 weeks Frequency: 5 days/week Volume: 40 min Intensity: 60–69% HR_peak_ EG: Mini-basketball program Activities: Start (lining up, roll call, and greetings), warm-up (light jogging, stretching, and general limb mobility), intervention—comprising three consecutive stages: (1) basic basketball training, (2) instruction in specific mini-basketball skills, and (3) a mini-basketball game—and cool-down. CON: Maintained their regular routine and usual care.	Social Responsiveness: Social Responsiveness Scale, Second Edition ■ Social awareness ■ Social cognition ■ Social communication ■ Social motivation ■ Behavior pattern	Intra-group (*p* < 0.05): ↓ Social cognition (score) in *EG* ↑ Social responsiveness (total score) in *CON* ↑ Social communication (score) in *CON* Inter-group (*p* < 0.05): *EG* < Social responsiveness (total score) than *CON* *EG* < Social communication (score) than *CON* *EG* < Social cognition (score) than *CON*	Dropouts: 29 lost to follow-up EG: 15 CON: 14 Adverse events: NR
Cai et al. (2019) [37] Country: China	Participants (*n*): 59 (30EG; 29CON) Final sample (*n*): 30 (15EG; 15CON) Sex: EG: 12M/3F CON: 14M/1F Age, *years* (mean; SD): EG: 5.03 ± 0.64 CON: 4.56 ± 0.84	Duration: 12 weeks Frequency: 5 days/week Volume: 40 min Intensity: NR EG: Mini-basketball program Activities: 5 min warm-up; (b) 20 min basic basketball skill instruction; (c) 10 min basketball game; (d) 5 min cool-down. CON: Maintained their regular routine.	Social Responsiveness: Social Responsiveness Scale, Second Edition ■ Social awareness ■ Social cognition ■ Social communication ■ Social motivation ■ Behavior pattern Physical Fitness Performances ■ 2 × 10 m shuttle run ■ Standing long jump ■ Sit-and-reach ■ Balance beam test	Intra-group (*p* < 0.05): ↓ Social responsiveness (total score) in *EG* ↓ Social cognition (score) in *EG* ↓ Social communication (score) in *EG* ↑ Social responsiveness (total score) in *CON* ↑ Social communication (score) in *CON* ↑ Behavior pattern (score) in *CON* ↓ 2 × 10 m shuttle run time in *EG* ↑ Standing long jump in *EG* Inter-group (*p* < 0.05): *EG* < Social responsiveness (total score) than *CON* *EG* < Social awareness (score) than *CON* *EG* < Social communication (score) than *CON* *EG* < Social cognition (score) than *CON* *EG* < Behavior pattern (score) than *CON* *EG* < 2 × 10 m shuttle run time than *CON* *EG* ˃ Standing long jump than *CON*	Dropouts: 29 lost to follow-up EG: 15 CON: 14 Adverse events: NR
Tse et al. (2019) [40] Country: China	Participants (*n*): 50 (25EG; 25CON) Final sample (*n*): 40 (19EG; 21CON) Sex: EG: 14M/5F CON: 18M/3F Age, *years* (mean; SD): EG: 10.11 ± 1.20 CON: 9.81 ± 1.17	Duration: 12 weeks Frequency: 2 days/week Volume: 44 min Intensity: NR EG: Basketball skill-learning intervention Activities: Warm-up (10 min); basketball instruction (30 min) to learn different basketball skills; cool-down (5 min). CON: Did not receive any intervention and were asked to follow their daily routine without taking part in any additional physical exercise program.	Self-reported sleep quality (parent-reported sleep log): Sleep duration (h) Sleep efficiency (%) Wake after sleep onset (min) Objective sleep status (actigraphy assessment): Sleep duration (h) Sleep efficiency (%) Wake after sleep onset (min) Neuropsychological measures: Inhibition control (false alarm error) Corsi block tapping task Forward digit span Backward digit span	Intra-group *(p < 0.05)* ↑ Sleep duration after intervention on weekday (actigraphy and sleep log) and weekend (actigraphy) in *EG* ↓ Sleep duration after intervention on weekday (actigraphy and sleep log) and weekend (sleep log) in *CON* ↑ Sleep efficiency after intervention on weekday and weekend in *EG* (actigraphy and sleep log) ↓ Sleep efficiency after intervention on weekday (actigraphy) and weekend (sleep log) in *CON* ↓ Wake after sleep onset after intervention on weekday and weekend in *EG* (actigraphy and sleep log) ↑ Wake after sleep onset after intervention on weekday in *CON* (actigraphy) ↓ Inhibition control (false alarm error) in *EG* Inter-group *(p < 0.05*) *EG* > Sleep duration compared to *CON* after intervention on weekday (actigraphy) and weekend (actigraphy and sleep log) *EG* > Sleep efficiency in compared to *CON* after intervention on weekday and weekend (actigraphy and sleep log) *EG* < Wake after sleep onset compared to *CON* after intervention on weekday and weekend (actigraphy and sleep log) *EG* < Inhibition control (false alarm error) than *CON*	Dropouts: 10 Due to incompletion of assessments EG: 6 CON: 4 Adverse events: NR

Note. >: greater; <: lower; ↑: increment; ↓: decrement; ABA: applied behavior analysis; AOI: areas of interests; CON: control group; EG: experimental group; F: female; M: male; NO: not observed; NR: not reported.

#### 3.5.2. Behavioral Outcome Measures

One study examined the Repetitive Behavior Scale [39]. Post-intervention, the mini-basketball group showed lower scores than the control group regarding the total score, self-injurious behavior, and restricted behavior. Within-group analyses indicated that the control group declined, with increases in total and restricted behavior.

#### 3.5.3. Joint Attention (Eye-Tracking)

One study measured joint attention metrics via eye-tracking [41]. In the control group, time to first fixation increased, and the number of accurate gaze shifts decreased. No statistically significant within-group changes were observed in the experimental group.

At post-test, the intervention group showed a greater area of interests fixation count, a shorter time to first fixation, more accurate gaze shifts, and a higher accurate-to-inaccurate gaze shift ratio than did the control group.

#### 3.5.4. Physical Fitness

Only one study evaluated physical fitness as the main outcome using four tests: the 2 × 10 m shuttle run, standing long jump, sit-and-reach, and balance beam [37]. The experimental group showed statistically significant within-group improvements, with a reduction in 2 × 10 m shuttle run time and an increase in standing long jump distance. In the between-group analysis, the experimental group achieved a shorter 2 × 10 m shuttle run time and a greater standing long jump distance than did the control group, whereas no significant differences were observed for the sit-and-reach or the balance beam tests.

#### 3.5.5. Neuropsychological Measures

The study conducted by Tse et al. [40] evaluated inhibition control (false alarm error), the Corsi block-tapping task, forward digit span, and backward digit span. Significant improvements were observed only in the basketball group for inhibition control (false alarm error), which also showed a greater reduction in errors compared to that of the control group. No significant differences were found for the remaining variables.

#### 3.5.6. Sleep Quality

One study evaluated sleep outcomes using actigraphy and parent-reported sleep logs [40]. Intra-group results showed that the basketball group showed improved sleep quality after the intervention (actigraphy and sleep log). Moreover, the experimental group showed better results than did the control group for the three variables analyzed (sleep duration, sleep efficiency, wake after sleep onset).

## 4. Discussion

Although involving children with ASD in team sports presents significant challenges, the evidence from this systematic review demonstrates that structured mini-basketball interventions can effectively address core symptoms and associated difficulties in this population. The aim of this review was to analyze the benefits of basketball for children with an ASD diagnosis based on RCTs, and our findings consistently show that mini-basketball programs improve social responsiveness, reduce behavioral symptoms, and enhance related outcomes across eight studies encompassing 436 participants. Five studies [34,35,36,37,38] demonstrated significant reductions in SRS-2 total scores following mini-basketball interventions, with effect sizes ranging from moderate to large. Specifically, Yang et al. [38] and Qi et al. [35] demonstrated that mini-basketball intervention or combining it with other ball training approaches yielded superior outcomes compared to those for the control groups, supporting recent meta-analytical evidence that PA interventions can improve core symptoms of ASD [9,16].

In the present systematic review, statistically significant improvements were observed in social communication and cognition. These findings appear to align with previous meta-analyses that have consistently identified mini-basketball as one of the potentially most effective forms of physical activity for enhancing social functioning in children with ASD [18,42]. Zhang et al. [39] demonstrated significant reductions in repetitive behaviors, self-injurious behaviors, and restricted behaviors compared to that of the controls. The social aspects of basketball may facilitate social behavior training and practice in more structured contexts (e.g., rule-following, cooperative gameplay), corroborated by meta-analytical evidence showing that PA can effectively reduce stereotypical behaviors in children with ASD [17]. In this sense, Ge et al. [41] found increased fixation counts, shorter time to first fixation, and more accurate gaze shifts in the mini-basketball training group. Also, the neuroplasticity effects observed by Yu et al. [36] and Yang et al. [34,38], who incorporated neuroimaging measures, provide insight into potential neural mechanisms underlying behavioral improvements, as exercise interventions can influence brain connectivity and activity patterns in children with ASD [3,4,14].

The intervention characteristics identified in this review show that all trials employed a 12-week duration and 40–45 min of PA per session [34,35,36,37,38,39,40,41]. While seven studies employed interventions of 5 days per week [34,35,36,37,38,39,41], Tse et al. [40] found that interventions of 2 days per week achieved significant improvements in sleep quality, inhibition control, and neuropsychological measures, suggesting that reduced frequency intervention may also be beneficial. Regarding activity intensity, the studies employed a moderate intensity (typically 128–148 bpm; 60–69% HRmax) [34,35,36,38,39,41] that appears to be well-tolerated and effective, aligning with previous findings that indicate periods of >8 weeks with moderate intensity demonstrate superior effects on various outcomes in children with ASD [13,15]. Cai et al. [37] only evaluated physical fitness parameters, demonstrating significant improvements in shuttle run times and standing long jump performance. Therefore, mini-basketball programs offer dual benefits for both motor and social development. In addition, the successful integration of basketball training with applied behavior analysis (ABA) services across all studies, as explicitly reported in the intervention descriptions, demonstrates practical feasibility within existing therapeutic frameworks and supports the potential for widespread implementation in clinical settings [20,21].

The comparison with other physical activities reveals that while martial arts often rank highest for stereotyped behavior outcomes, basketball interventions offer unique advantages in terms of accessibility, scalability, and integration into school-based programs [11,26]. Qi et al. [35] directly compared mini-basketball with a ball-combination program that progressed from basketball to soccer, finding that both interventions achieved significant improvements. This suggests that basketball’s benefits may be maintained even when combined with other ball sports, potentially offering programming flexibility. The motor skill requirements identified across studies—from basic dribbling and shooting in early phases to complex game scenarios—may serve as a basis for broader social and behavioral improvements. In this sense, meta-analytical evidence shows that exercise interventions can significantly improve motor outcomes in children with ASD [10,19,43]. The multi-domain benefits observed across studies, from Tse et al.’s [40] sleep improvements (increased duration and efficiency, reduced wake after sleep onset) to the neuropsychological enhancements (better inhibition control), physical fitness gains [37], joint attention improvements [41], and behavioral symptom reductions [39], suggest that basketball interventions provide comprehensive therapeutic effects beyond the primary social responsiveness outcomes [34,35,36,37,38].

Although this review demonstrates originality, several limitations should be taken into account when interpreting the findings. First, all included studies were conducted in China and may have been produced by the same research team, introducing potential risks of sample non-independence and geographic bias. These factors limit the generalizability of the results to other sociocultural contexts. Differences in cultural norms, educational systems, and patterns of physical activity participation may influence how children with ASD engage in team-based sports and respond socially. Second, the follow-up periods across the included studies were generally short, making it difficult to draw firm conclusions regarding the long-term maintenance of intervention benefits. Additionally, several studies reported high dropout rates, and most failed to document adverse events, thereby hindering a comprehensive assessment of adherence and the safety profile of the mini-basketball intervention. Furthermore, the number of included randomized controlled trials was limited. Only two studies were rated as showing “good” quality, while the remainder were classified as “fair,” indicating that the overall level of evidence remains moderate. Common methodological limitations included the absence of blinding procedures, inadequate sample size planning, and incomplete outcome data. Some studies also exhibited potential sample overlap and heterogeneity in outcome measures, which restricted the feasibility of conducting a meta-analysis.

To address these limitations, future research should aim to replicate studies across diverse cultural and social settings to evaluate the universality and scalability of mini-basketball interventions. Longer follow-up periods are needed to assess the sustainability of intervention effects over time. In addition, the inclusion of more diverse participant samples—such as children with ASD of different genders, age groups, and functional levels—will improve the representativeness and external validity of the findings. Moreover, future trials should examine how variations in team composition (e.g., mixed teams of children with and without ASD) and multi-sport combination programs influence intervention outcomes in order to further elucidate the mechanisms by which organized physical activity mediated through ball sports contributes to improvements in core ASD symptoms.

## 5. Conclusions

Mini-basketball interventions may be associated with improvements in core symptoms and related difficulties among preschool and school-aged children with ASD. In the available findings, significant improvements in social responsiveness were observed, with results generally exceeding those of the control groups. Additionally, some studies reported improvements in behavioral symptoms, joint attention, physical fitness, neuropsychological measures, and sleep quality.

The intervention parameters used recurrently (12 weeks in duration, 40–45 min per session, predominantly 5 days per week at moderate intensity) may represent a viable option for achieving clinically meaningful improvements; however, further studies with longer durations and varying intensities are warranted. The results of this review also suggest that mini-basketball may be a feasible, safe, and potentially effective adjunctive intervention that can be integrated with existing therapeutic approaches such as applied behavior analysis (ABA). Given that all current evidence originates from studies conducted in China, the applicability of these findings to other cultural and social contexts should be interpreted with caution.

Based on these findings, some practical applications can be considered with appropriate caution. Mini-basketball programs appear advantageous because they require minimal equipment, follow a structured format, and can be adapted to diverse settings. The parameters supported by the available evidence provide preliminary guidance for program design, and integration with ABA services suggests compatibility with established therapeutic frameworks. Consequently, health and education professionals might consider incorporating structured mini-basketball programs into comprehensive intervention plans, particularly when social communication limitations are present.

## Figures and Tables

**Table 1:** Full search strategy for each database, presented as they were used.

Data Base	Search Strategy	Results
Web Of Science	TS ((“basketball” OR “mini-basketball”) AND (“autis*” OR “autism spectrum disorder” OR “ASD” OR “autist*”))	44
Scopus	TITLE-ABS-KEY ((“basketball” OR “mini-basketball”) AND (“autis*” OR “autism spectrum disorder” OR “ASD” OR “autist*”))	49
SPORTDiscus	(“basketball” OR “mini-basketball”) AND (“autis*” OR “autism spectrum disorder” OR “ASD” OR “autist*”)	32
MEDLINE/ PubMed	(“basketball” OR “mini-basketball”) AND (“autis*” OR “autism spectrum disorder” OR “ASD” OR “autist*”)	25
TOTAL		150

**Table 2:** Quality assessment of randomized controlled trials.

Authors (Year)	Items												Quality Rating
	1	2	3	4	5	6	7	8	9	10	11	Score	
Yang et al. (2024) [34]	Y	+	−	+	−	−	−	−	−	+	+	4/10	Fair
Qi et al. (2024) [35]	Y	+	−	+	−	−	+	−	−	+	+	5/10	Fair
Yu et al. (2021) [36]	Y	+	−	+	−	−	−	−	−	+	+	4/10	Fair
Cai et al. (2019) [37]	Y	+	−	+	−	−	−	−	−	+	+	4/10	Fair
Yang et al. (2021) [38]	Y	+	−	+	−	−	−	−	−	+	+	4/10	Fair
Zhang et al. (2025) [39]	Y	+	−	+	−	−	−	−	−	+	+	4/10	Fair
Tse et al. (2019) [40]	Y	+	−	+	−	−	+	+	−	+	+	6/10	Good
Ge et al. (2024) [41]	Y	+	+	+	−	−	+	−	−	+	+	6/10	Good

Note. Eligibility criteria item does not contribute to total score. Items: (1) eligibility criteria; (2) randomization; (3) concealed allocation; (4) similarity at baseline; (5) subjects blinding; (6) therapist blinding; (7) assessor blinding; (8) one key outcome measured in >85% of subjects; (9) intention-to-treat analysis; (10) between-group statistical results for one key outcome; (11) measures of variability and point measures for one key outcome. “+” indicates that the criterion was met; “−” indicates that the criterion was not met.

## Data Availability

No new data were created or analyzed in this study. Data sharing is not applicable to this article.

## Supplementary Materials

The following supporting information can be downloaded at: https://www.mdpi.com/article/10.3390/healthcare13222861/s1, File S1: The PRISMA 2020 statement: an updated guideline for reporting systematic reviews.

## References

[1] American Psychiatric Association (2013). Diagnostic and Statistical Manual of Mental Disorders.

[2] Wilson A.C. (2024). Cognitive Profile in Autism and ADHD: A Meta-Analysis of Performance on the WAIS-IV and WISC-V. Arch. Clin. Neuropsychol..

[3] Donovan A.P.A., Basson M.A. (2017). The Neuroanatomy of Autism—A Developmental Perspective. J. Anat..

[4] O’Reilly C., Lewis J.D., Elsabbagh M. (2017). Is Functional Brain Connectivity Atypical in Autism? A Systematic Review of EEG and MEG Studies. PLoS ONE.

[5] Zhou M.S., Nasir M., Farhat L.C., Kook M., Artukoglu B.B., Bloch M.H. (2021). Meta-Analysis: Pharmacologic Treatment of Restricted and Repetitive Behaviors in Autism Spectrum Disorders. J. Am. Acad. Child Adolesc. Psychiatry.

[6] Alfageh B.H., Wang Z., Mongkhon P., Besag F.M.C., Alhawassi T.M., Brauer R., Wong I.C.K. (2019). Safety and Tolerability of Antipsychotic Medication in Individuals with Autism Spectrum Disorder: A Systematic Review and Meta-Analysis. Pediatr. Drugs.

[7] Salazar De Pablo G., Pastor Jordá C., Vaquerizo-Serrano J., Moreno C., Cabras A., Arango C., Hernández P., Veenstra-VanderWeele J., Simonoff E., Fusar-Poli P. (2023). Systematic Review and Meta-Analysis: Efficacy of Pharmacological Interventions for Irritability and Emotional Dysregulation in Autism Spectrum Disorder and Predictors of Response. J. Am. Acad. Child Adolesc. Psychiatry.

[8] Chen Y.-C.B., Lin H.-Y., Wang L.-J., Hung K.-C., Brunoni A.R., Chou P.-H., Tseng P.-T., Liang C.-S., Tu Y.-K., Lin P.-Y. (2024). A Network Meta-Analysis of Non-Invasive Brain Stimulation Interventions for Autism Spectrum Disorder: Evidence from Randomized Controlled Trials. Neurosci. Biobehav. Rev..

[9] Huang J., Du C., Liu J., Tan G. (2020). Meta-Analysis on Intervention Effects of Physical Activities on Children and Adolescents with Autism. IJERPH.

[10] Healy S., Nacario A., Braithwaite R.E., Hopper C. (2018). The Effect of Physical Activity Interventions on Youth with Autism Spectrum Disorder: A Meta-analysis. Autism Res..

[11] Monteiro C.E., Da Silva E., Sodré R., Costa F., Trindade A.S., Bunn P., Costa E Silva G., Di Masi F., Dantas E. (2022). The Effect of Physical Activity on Motor Skills of Children with Autism Spectrum Disorder: A Meta-Analysis. Int. J. Environ. Res. Public Health.

[12] Liang X., Haegele J.A., Healy S., Tse A.C.-Y., Qiu H., Zhao S., Li C. (2023). Age-Related Differences in Accelerometer-Assessed Physical Activity and Sleep Parameters Among Children and Adolescents With and Without Autism Spectrum Disorder: A Meta-Analysis. JAMA Netw. Open.

[13] Liang X., Haegele J.A., Tse A.C.-Y., Li M., Zhang H., Zhao S., Li S.X. (2024). The Impact of the Physical Activity Intervention on Sleep in Children and Adolescents with Autism Spectrum Disorder: A Systematic Review and Meta-Analysis. Sleep Med. Rev..

[14] Tao R., Yang Y., Wilson M., Chang J.R., Liu C., Sit C.H.P. (2025). Comparative Effectiveness of Physical Activity Interventions on Cognitive Functions in Children and Adolescents with Neurodevelopmental Disorders: A Systematic Review and Network Meta-Analysis of Randomized Controlled Trials. Int. J. Behav. Nutr. Phys. Act..

[15] Sung M.-C., Ku B., Leung W., MacDonald M. (2022). The Effect of Physical Activity Interventions on Executive Function Among People with Neurodevelopmental Disorders: A Meta-Analysis. J. Autism Dev. Disord..

[16] Wang S., Chen D., Yang Y., Zhu L., Xiong X., Chen A. (2023). Effectiveness of Physical Activity Interventions for Core Symptoms of Autism Spectrum Disorder: A Systematic Review and Meta-analysis. Autism Res..

[17] Ferreira J.P., Ghiarone T., Cabral Júnior C.R., Furtado G.E., Moreira Carvalho H., Machado-Rodrigues A.M., Andrade Toscano C.V. (2019). Effects of Physical Exercise on the Stereotyped Behavior of Children with Autism Spectrum Disorders. Medicina.

[18] Kou R., Li Z., Li M., Zhou R., Zhu F., Ruan W., Zhang J. (2024). Comparative Effectiveness of Physical Exercise Interventions on Sociability and Communication in Children and Adolescents with Autism: A Systematic Review and Network Meta-Analysis. BMC Psychol..

[19] Rosales M.R., Butera C.D., Wilson R.B., Zhou J., Maus E., Zhao H., Chow J.C., Dao A., Freeman J., Dusing S.C. (2025). Systematic Review and Meta-Analysis of the Effect of Motor Intervention on Cognition, Communication, and Social Interaction in Children with Autism Spectrum Disorder. Phys. Occup. Ther. Pediatr..

[20] He J., Gong Y., Yin M., Zhang L., Wu X. (2025). Optimal Dosage of Group-Based Organized Physical Activity for Enhancing Social Abilities in Autistic Children: Insights from a Multilevel Meta-Analysis. Int. J. Behav. Nutr. Phys. Act..

[21] Howells K., Sivaratnam C., May T., Lindor E., McGillivray J., Rinehart N. (2019). Efficacy of Group-Based Organised Physical Activity Participation for Social Outcomes in Children with Autism Spectrum Disorder: A Systematic Review and Meta-Analysis. J. Autism Dev. Disord..

[22] O’Flahert M., Hill J., Bourke M., Gomersall L., Tweedy S., Cairney J. (2025). Comparing Trajectories of Sport Participation for Autistic- and Non-Autistic-Youth: A Group-Based Multi-Trajectory Modelling Approach. Autism.

[23] Vetri L., Roccella M. (2020). On the Playing Field to Improve: A Goal for Autism. Medicina.

[24] Howells K., Sivaratnam C., Lindor E., Hyde C., McGillivray J., Whitehouse A., Rinehart N. (2020). Can Participation in a Community Organized Football Program Improve Social, Behavioural Functioning and Communication in Children with Autism Spectrum Disorder? A Pilot Study. J. Autism Dev. Disord..

[25] Howells K., Sivaratnam C., Lindor E., He J., Hyde C., McGillivray J., Wilson R.B., Rinehart N. (2022). Can a Community-Based Football Program Benefit Motor Ability in Children with Autism Spectrum Disorder? A Pilot Evaluation Considering the Role of Social Impairments. J. Autism Dev. Disord..

[26] Hou Y., Wang Y., Deng J., Song X. (2024). Effects of Different Exercise Interventions on Executive Function in Children with Autism Spectrum Disorder: A Network Meta-Analysis. Front. Psychiatry.

[27] Stachura K., Emich-Widera E., Kazek B., Stania M. (2025). Coordination, Balance and Fine Motor Skills Deficities in Children with Autism Spectrum Disorder Without Co-Occuring Conditions-Application of MABC-2 Test in Pilot Study Among Polish Children. J. Clin. Med..

[28] Wang J.-G., Cai K.-L., Liu Z.-M., Herold F., Zou L., Zhu L.-N., Xiong X., Chen A.-G. (2020). Effects of Mini-Basketball Training Program on Executive Functions and Core Symptoms among Preschool Children with Autism Spectrum Disorders. Brain Sci..

[29] Fotrousi F., Bagherly J., Ghasemi A. (2012). The Compensatory Impact of Mini-Basketball Skills on the Progress of Fundamental Movements in Children. Procedia—Soc. Behav. Sci..

[30] Page M.J., McKenzie J.E., Bossuyt P.M., Boutron I., Hoffmann T.C., Mulrow C.D., Shamseer L., Tetzlaff J.M., Akl E.A., Brennan S.E. (2021). The PRISMA 2020 Statement: An Updated Guideline for Reporting Systematic Reviews. BMJ.

[31] Rico-González M., Pino-Ortega J., Clemente F.M., Los Arcos A. (2022). Guidelines for Performing Systematic Reviews in Sports Science. Biol. Sport..

[32] Ouzzani M., Hammady H., Fedorowicz Z., Elmagarmid A. (2016). Rayyan-a Web and Mobile App for Systematic Reviews. Syst. Rev..

[33] Silverman S.R., Schertz L.A., Yuen H.K., Lowman J.D., Bickel C.S. (2012). Systematic Review of the Methodological Quality and Outcome Measures Utilized in Exercise Interventions for Adults with Spinal Cord Injury. Spinal Cord.

[34] Yang Y., Chen D., Cai K., Zhu L., Shi Y., Dong X., Sun Z., Qiao Z., Yang Y., Zhang W. (2024). Effects of Mini-Basketball Training Program on Social Communication Impairments and Regional Homogeneity of Brain Functions in Preschool Children with Autism Spectrum Disorder. BMC Sports Sci. Med. Rehabil..

[35] Qi K., Liu Y., Wang Z., Xiong X., Cai K., Xu Y., Shi Y., Sun Z., Dong X., Chen A. (2024). Recreational Ball Games Are Effective in Improving Social Communication Impairments among Preschoolers Diagnosed with Autism Spectrum Disorder: A Multi-Arm Controlled Study. BMC Sports Sci. Med. Rehabil..

[36] Yu H., Qu H., Chen A., Du Y., Liu Z., Wang W. (2021). Alteration of Effective Connectivity in the Default Mode Network of Autism After an Intervention. Front. Neurosci..

[37] Cai K.L., Wang J.G., Liu Z.M., Zhu L.N., Xiong X., Klich S., Maszczyk A., Chen A.G. (2020). Mini-Basketball Training Program Improves Physical Fitness and Social Communication in Preschool Children with Autism Spectrum Disorders. J. Hum. Kinet..

[38] Yang S., Liu Z., Xiong X., Cai K., Zhu L., Dong X., Wang J., Zhu H., Shi Y., Chen A. (2021). Effects of Mini-Basketball Training Program on Social Communication Impairment and Executive Control Network in Preschool Children with Autism Spectrum Disorder. Int. J. Environ. Res. Public Health.

[39] Zhang W., Cai K., Xiong X., Zhu L., Sun Z., Yang S., Cheng W., Mao H., Chen A. (2025). Alterations of Triple Network Dynamic Connectivity and Repetitive Behaviors after Mini-Basketball Training Program in Children with Autism Spectrum Disorder. Sci. Rep..

[40] Tse C.Y.A., Lee H.P., Chan K.S.K., Edgar V.B., Wilkinson-Smith A., Lai W.H.E. (2019). Examining the Impact of Physical Activity on Sleep Quality and Executive Functions in Children with Autism Spectrum Disorder: A Randomized Controlled Trial. Autism.

[41] Ge L.K., Man X., Cai K., Liu Z., Tsang W.W., Chen A., Wei G.X. (2024). Sharing Our World: Impact of Group Motor Skill Learning on Joint Attention in Children with Autism Spectrum Disorder. J. Autism Dev. Disord..

[42] Wang Y., Qian G., Mao S., Zhang S. (2025). The Impact of Physical Exercise Interventions on Social, Behavioral, and Motor Skills in Children with Autism: A Systematic Review and Meta-Analysis of Randomized Controlled Trials. Front. Pediatr..

[43] Ruggeri A., Dancel A., Johnson R., Sargent B. (2020). The Effect of Motor and Physical Activity Intervention on Motor Outcomes of Children with Autism Spectrum Disorder: A Systematic Review. Autism.

